# The Epidermal Growth Factor Ligand Spitz Modulates Macrophage Efferocytosis, Wound Responses and Migration Dynamics During *Drosophila* Embryogenesis

**DOI:** 10.3389/fcell.2021.636024

**Published:** 2021-04-08

**Authors:** Olivier R. Tardy, Emma L. Armitage, Lynne R. Prince, Iwan R. Evans

**Affiliations:** ^1^Department of Infection, Immunity and Cardiovascular Disease, The Bateson Centre, The University of Sheffield, Sheffield, United Kingdom; ^2^The Bateson Centre, The University of Sheffield, Sheffield, United Kingdom

**Keywords:** *Drosophila*, macrophage, hemocyte, epidermal growth factor, cell migration, inflammation, apoptotic cell clearance

## Abstract

How multifunctional cells such as macrophages interpret the different cues within their environment and undertake an appropriate response is a key question in developmental biology. Understanding how cues are prioritized is critical to answering this – both the clearance of apoptotic cells (efferocytosis) and the migration toward damaged tissue is dependent on macrophages being able to interpret and prioritize multiple chemoattractants, polarize, and then undertake an appropriate migratory response. Here, we investigate the role of Spitz, the cardinal *Drosophila* epidermal growth factor (EGF) ligand, in regulation of macrophage behavior in the developing fly embryo, using activated variants with differential diffusion properties. Our results show that misexpression of activated Spitz can impact macrophage polarity and lead to clustering of cells in a variant-specific manner, when expressed either in macrophages or the developing fly heart. Spitz can also alter macrophage distribution and perturb apoptotic cell clearance undertaken by these phagocytic cells without affecting the overall levels of apoptosis within the embryo. Expression of active Spitz, but not a membrane-bound variant, can also increase macrophage migration speeds and impair their inflammatory responses to injury. The fact that the presence of Spitz specifically undermines the recruitment of more distal cells to wound sites suggests that Spitz desensitizes macrophages to wounds or is able to compete for their attention where wound signals are weaker. Taken together these results suggest this molecule regulates macrophage migration and their ability to dispose of apoptotic cells. This work identifies a novel regulator of *Drosophila* macrophage function and provides insights into signal prioritization and integration *in vivo*. Given the importance of apoptotic cell clearance and inflammation in human disease, this work may help us to understand the role EGF ligands play in immune cell recruitment during development and at sites of disease pathology.

## Introduction

Understanding how motile cells integrate and prioritize the array of signals they face in the complex *in vivo* environment is a fundamental question in biology. For multifunctional cells such as macrophages this is a particularly important process, since it determines their subsequent behavior, be it migration to sites of damage, or clearance of pathogens and dying cells. The integration of a specific cue is highly contextual and depends on a number of parameters including crosstalk between signal transduction pathways (Heit et al., 2008), calcium levels within cells (Dou et al., 2012; Sieger et al., 2012), and the diffusion properties of a given ligand (Foxman et al., 1997). However, even before we are able to understand how cells prioritize different cues *in vivo*, and so are able to polarize and migrate toward their correct targets, it is necessary to identify a more complete range of cues to which they can respond.

*Drosophila melanogaster* fruit flies have a robust cellular immune response, composed principally of motile and highly-phagocytic plasmatocytes (Evans and Wood, 2011), which perform many analogous functions to vertebrate macrophages, e.g., phagocytosis of apoptotic cells and pathogens, migration to wounds and secretion of extracellular matrix (Buchon et al., 2014; Weavers et al., 2016). These cells (referred to hereafter as embryonic macrophages) have been extensively used to investigate cell polarization and migration *in vivo*, although we are yet to understand the full complement of cues that regulate their behaviors. Post hematopoiesis, embryonic *Drosophila* macrophages undertake stereotypical patterns of dispersal across the embryo. This dispersal is governed by PDGF/Vegf-related ligands (Pvfs) that act both as chemoattractants and pro-survival signals (Cho et al., 2002; Brückner et al., 2004; Wood et al., 2006), cell–cell repulsion between macrophages (Davis et al., 2012), and access to physical spaces created during organogenesis (Evans et al., 2010a). Once dispersed over the embryo (stage 15 onwards), macrophages become competent to respond to wounding stimuli (Moreira et al., 2010) and undergo “random migration,” a process driven in part by cell–cell repulsion (Stramer et al., 2010). Alongside deposition of matrix during dispersal (Matsubayashi et al., 2017), clearance of apoptotic cells (efferocytosis) and responses to acute wound stimuli represent migration-dependent functions that require polarization and migration of these macrophages toward specific targets. Apoptosis is the major form of programmed cell death in multicellular organisms (Fuchs and Steller, 2011; Galluzzi et al., 2012) and rapid efferocytosis is required to prevent secondary necrosis, a highly pro-inflammatory event that can lead to subsequent tissue damage (Degterev and Yuan, 2008; Ariel and Ravichandran, 2016). Failures in efferocytosis are linked to a range of disease pathologies in humans, particularly those associated with chronic inflammation, including atherosclerosis and chronic obstructive pulmonary disease (Eltboli et al., 2014; Morioka et al., 2019). The recruitment of macrophages to apoptotic cells is mediated by a family of chemoattractants released as part of the apoptotic cell death program and collectively referred to as “find-me” cues (Ravichandran, 2003). While find-me cues have been extensively studied in mammals, e.g., lysophosphatidylcholine (LPC) or ATP (Lauber, 2003; Elliott et al., 2009), the identity of such signals remains unknown in *Drosophila*, although fragments of tyrosyl tRNA synthetase have been shown to play a role in recruitment of macrophages to apoptotic “loser” cells in studies of cell competition (Casas-Tintó et al., 2015). *Drosophila* embryonic macrophages also undertake polarized migration when responding to tissue damage (Stramer et al., 2005). This process requires the generation of reactive oxygen species (Razzell et al., 2013; Evans et al., 2015), resembling inflammatory responses in other model organisms, including zebrafish (Niethammer et al., 2009; Yoo et al., 2011). As per find-me cues, the precise nature of wound cues remains to be elucidated in flies.

Recent evidence in *Drosophila* suggested that an epidermal growth factor (EGF) ligand homolog, Spitz, is secreted from midgut cells undergoing apoptosis. This facilitates recruitment of stem cells to replenish the cells in those apoptotic regions, thereby maintaining gut integrity (Liang et al., 2017). A chemoattractive role for EGF ligands is conserved across evolution with, amongst others, both human monocytes and border cells in the developing *Drosophila* oocyte shown to chemotax toward EGF ligands (Duchek and Rørth, 2001; Lamb et al., 2004). In contrast to mammalian EGF receptor signaling, which is composed of multiple heterodimeric ErbB receptors and ligands (Burgess et al., 2003; Citri and Yarden, 2006), *Drosophila* possess only a single EGF receptor (EGFR/Torpedo). EGFR is activated by several partially redundant ligands (Spitz, Vein, Keren, and Gurken) that are expressed in a tissue-specific manner (Price et al., 1989). In both flies and humans, secretion of active EGF ligands is tightly regulated via activation of the proteolytic enzymes Rhomboid (Shilo, 2016) and ADAM17 (Scheller et al., 2011; Rose-John, 2013), respectively. During *Drosophila* development, Spitz is ubiquitously expressed. However, the key processing enzyme Rhomboid is expressed in a tissue-specific pattern, including by the cells of the ventral midline (Tomancak et al., 2007; Frise et al., 2010). This post-translational control enables spatial specificity of action, for instance the role of Spitz in development of the midline glia (Raz and Shilo, 1992). The combined evolutionary and developmental evidence suggested to us that Spitz might have a role as a chemoattractant regulating *Drosophila* macrophage behavior.

In this study, we have used tissue-specific expression of two active variants of the EGF ligand Spitz to investigate how high levels of EGF signaling can alter the migration and function of *Drosophila* macrophages *in vivo*. Our results show that expression of active Spitz in macrophages alters their migration dynamics, increasing migration speed and stimulating macrophage clustering and elongation. These phenotypes require cleavage of Spitz from the membrane since a membrane-bound variant does not alter macrophage behavior. In addition, our results show that the presence of Spitz reduces the sensitivity of macrophages to both apoptotic and wound-derived signals. Our results demonstrate the capacity for EGF signaling to regulate diverse and important macrophage behaviors *in vivo* and suggest the possibility that EGF ligands may belong to a growing list of apoptotic cell-derived, find-me cues. These effects of EGF signaling on efferocytosis, inflammatory responses and macrophage migration have implications for our understanding of macrophage function during both embryogenesis and chronic inflammation, where these ligands play important roles in development and at sites of pathology in higher organisms.

## Materials and Methods

### Fly Stocks

Fly stocks were maintained on molasses-based media supplemented with yeast at 18°C with mating populations kept in laying cages at 22°C. Embryos were collected from apple juice/agar plates on which embryos had been laid overnight. The following *Drosophila* lines were used in this study: *UAS-EGFP*, *UAS-red stinger* (Barolo et al., 2000), *UAS-sSpitz^*CS*^* (Ghiglione et al., 2002), *UAS-Spitz^*Sec*^* (Miura et al., 2006), *UAS-mSpitz^*CS*^-EGFP* (Miura et al., 2006), *UAS-LifeAct* (Hatan et al., 2011), *Srp-3x-mCherry* (Gyoergy et al., 2018), *EGFR-sfGFP* (Revaitis et al., 2020), *Serpent-GAL4* (Brückner et al., 2004), *Croquemort-GAL4* (Stramer et al., 2005), and *TinC-GAL4* (Lo and Frasch, 2001). All experiments were conducted on a *w*^1118^ background. See Supplementary Table 1 for specific experimental genotypes.

### Preparation, Imaging and Wounding of Live Embryos

Embryos laid on apple juice/agar plates were washed off into a cell strainer and dechorionated in 5% bleach for 1 min, followed by five washes in distilled water. Embryos were mounted in 10S Voltalef oil (VWR) as per Evans et al., 2010b. Live embryos were imaged using a Perkin Elmer Ultraview Spinning disk system using either a 10× air (UplanSApo 10×/NA 0.4; lateral images of stage 15 embryos to show developmental dispersal of macrophages or to quantify total number of macrophages per embryo) or 40× oil immersion (UplanSApo 40× oil/NA 1.3; all remaining live imaging) objective lens. For analysis of macrophage random migration and wound responses, the ventral surface of stage 15 embryos was imaged to a depth of approximately 20 μm with a 1 μm spacing between *z*-planes. Time-lapse movies were assembled from *z*-stacks taken every 2 min for 1 h using Volocity software (Perkin Elmer) for analysis of both macrophage random migration and wound responses. Wounding was performed using a Micropoint ablation laser (Andor) to ablate the ventral epithelium on the ventral midline in the medial-most segments of the embryo as per Evans et al. (2015); the inflammatory responses of macrophages in this region were then followed for 1-h post wounding.

### Immunostaining of *Drosophila* Embryos

Live embryos were fixed as previously described (Wood et al., 2006). For immunostaining, dechorionated embryos were fixed using a 50:50 mixture of 4% formaldehyde in phosphate-buffered saline (PBS; Oxoid) and peroxide-free heptane (Sigma) before being devitellinised using methanol. Embryos were then washed with 0.1% Triton-X-100 (Sigma) in PBS. Subsequently, embryos were blocked in PATx [1% Bovine Serum Albumin (Sigma), 0.1% Triton-X-100 in PBS] for 1 h. Embryos were then incubated with primary antibodies overnight at 4°C, washed in PATx and incubated with secondary antibodies for 2 h at room temperature. After a final series of PATx washes, residual PATx was aspirated and the embryos stored at 4°C in 2.5% 1,4 Diazabicyclo[2.2.2]octane (DABCO) mountant (Sigma) diluted in 90% glycerol (Sigma)/1× PBS. Stained embryos were mounted in DABCO mountant on glass slides as per Evans et al., 2010a. Images of immunostained embryos were taken using a Zeiss 880 Airyscan confocal microscopy system running ZEN software. Embryos were imaged using a 40× objective lens (Zeiss Plan-Apochromat 40× oil/NA 1.4) to a depth of approximately 25 μm with a spacing of 0.2 μm between *z*-planes (cDCP-1 and DpERK staining) or using a 63× objective lens (Zeiss Plan-Apochromat 63× oil/NA 1.4; EGFR-sfGFP localization). For staining of apoptotic cells and GFP (expressed in macrophages to enable their visualization), the following primary antibodies were used: rabbit anti-cleaved DCP-1 (cDCP-1; 1:200; 9578S, Cell Signaling) and mouse anti-GFP (1:100; ab1218, Abcam). As a read-out of EGFR activation in macrophages, embryos were stained for activated ERK [DpERK; rabbit anti-phospho-p44/42 MAPK (Erk1/2) (Thr202/Tyr204); 1:100; 197G2, Cell Signaling Technology] with GFP-labeled macrophages detected via immunostaining for GFP (1:100; ab1218, Abcam). AlexaFluor568 goat anti-rabbit IgG (1:200; A11036, Life Technologies) and AlexaFluor488 goat anti-mouse IgG (1:200; A11029, Life Technologies) were used as secondary antibodies to detect anti-GFP, anti-cDCP-1 and anti-DpERK primary antibodies. To detect EGFR-sfGFP and macrophages, respectively, Rabbit anti-GFP (1:500; ab290, Abcam) and mouse anti-Fascin (purified sn 7C antibody diluted 1:1000; Developmental Studies Hybridoma Bank) were used as primary antibodies. AlexaFluor488 goat anti-rabbit IgG (1:200; A11034, Life Technologies) and AlexaFluor568 goat anti-mouse IgG (1:200; A11031, Life Technologies) were used as secondary antibodies.

### Lysotracker Red Staining of Embryos

pH-sensitive Lysotracker Red DND-99 (L7528, Life Technologies) was used to monitor acidification of phagosomes. Dechorionated embryos were transferred to glass vials containing peroxide-free heptane and PBS containing lysotracker red (25 μM) in a 1:1 ratio and shaken for 30 min at 250 rpm in the dark. Post staining, embryos were transferred into Halocarbon oil 700 (Sigma); stage 15 embryos were selected and the ventral midline region imaged using a Perkin Elmer Ultraview Spinning disk system (UplanSApo 40× oil objective lens/NA 1.3).

### Image Processing and Analysis

Images were converted to Tiff (.tif) format files prior to analysis in Fiji (ImageJ; Schindelin et al., 2012). Movies and stills showing macrophage morphology, apoptotic cell clearance, migration and lateral views of stage 15 embryos were assembled as maximum projections and despeckled to reduce background noise. Clustering of macrophages was assessed by counting the number of macrophage-macrophage contacts from maximum projections of the ventral midline region at stage 15 in Fiji. Only definite contacts between the cell bodies of neighboring macrophages were scored. Morphological parameters (e.g., aspect ratio (AR), which is defined as the ratio of a cell’s width to its height) were measured manually from maximum projections using the polygon selection tool in Fiji. Macrophage vacuolation, a read-out of apoptotic cell clearance, was assessed in the z-slice corresponding to that cell’s largest cross-sectional area in 5 macrophages per embryo. Apoptotic cell clearance was also analyzed using embryos containing GFP-labeled macrophages immunostained for cDCP-1 and GFP. The numbers of cDCP-1-positive punctae inside (within GFP-positive cell areas) or outside macrophages in a field of view corresponding to the medial-most ventral region of stage 15 embryos were counted in merged *z*-stacks of the GFP and cDCP-1 channels. These values were used to calculate the total numbers of cDCP-1 punctae and “efferocytosis efficiency” per field of view. Efferocytosis efficiency was defined as the percentage of the total numbers of cDCP-1 punctae engulfed by macrophages within the field of view, normalized according to numbers of macrophages within that field of view. Numbers of lysotracker-positive vacuoles per macrophage were counted from *z*-stacks of the ventral midline region; volumes of individual lysotracker-positive vacuoles were analyzed using Imaris software (Oxford Instruments). Quantification of DpERK levels within macrophages on the ventral surface of the embryo was carried out using IMARIS Surpass 3D rendering software. GFP staining was used to mask macrophages and measure total DpERK intensity per cell. Total intensity per cell was then divided by the volume of each macrophage (μm^3^), with 15–20 macrophages per embryo quantified. These values were then averaged per embryo.

The Fiji manual tracking plug-in was used to track cell movements of macrophages undergoing random migration from the assembled time-lapse movies. Tracking data was then imported into the Ibidi Chemotaxis tool plugin in Fiji to calculate migratory parameters (Petrie et al., 2009).

**FIGURE 1:**
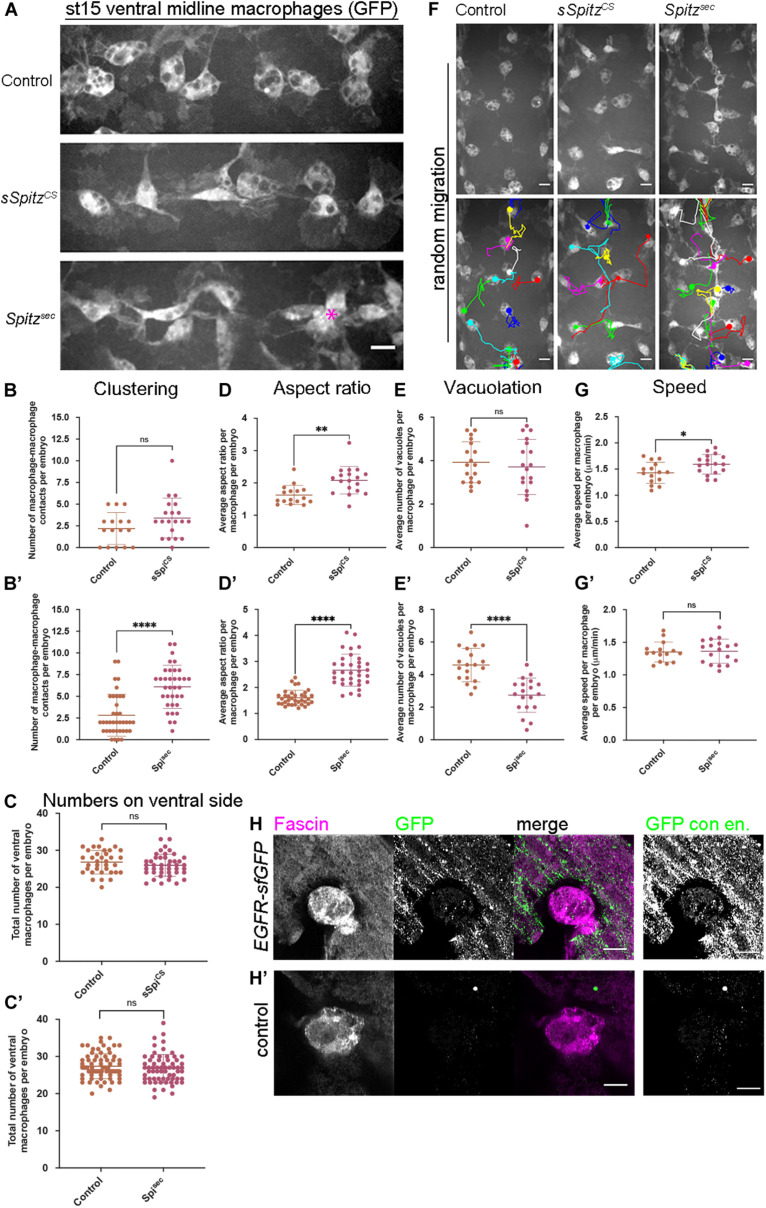
Spitz stimulates macrophage polarization, impairs efferocytosis and alters migration dynamics. **(A)** Maximum projections of GFP-tagged macrophages on the ventral midline at stage 15 in control embryos and in embryos with macrophage-specific expression of sSpitz^CS^ or Spitz^sec^; asterisk (^∗^) shows cluster of macrophages on midline; anterior is left. **(B,B′)** Scattergraphs showing degree of macrophage clustering via number of macrophage-macrophage contacts per embryo in the presence of *sSpitz*^*CS*^ **(B)** (*n* = 16, 20; *p* = 0.0977) or Spitz^sec^ **(B′)** (*n* = 38, 35; *p* < 0.0001). **(C,C′)** Scattergraphs showing number of macrophages in the ventral midline region (VML) per embryo at stage 15 in the presence of *sSpitz*^*CS*^ **(C)** (*n* = 35, 43; *p* = 0.229) or *Spitz*^*Sec*^ (**C′,D**) (*n* = 73, 65; *p* = 0.247). **(D,D′)** Scattergraphs showing aspect ratio per macrophage per embryo in the presence of *sSpitz*^*CS*^ **(D)** (*n* = 16, 18; *p* = 0.0018) or *Spitz*^*Sec*^ **(D′)** (*n* = 35, 34; *p* < 0.0001). **(E,E′)** Scattergraphs showing average numbers of vacuoles per macrophage per embryo in controls in the presence of *sSpitz*^*CS*^ **(E)** (*n* = 18, 18; *p* = 0.574) or *Spitz*^*sec*^ **(E′)** (*n* = 19, 19; *p* < 0.0001). **(F)** Maximum projections and macrophage tracking data of GFP-labeled macrophages on the ventral midline at stage 15 in control embryos and in embryos with macrophage-specific expression of sSpitz^CS^ or Spitz^sec^; anterior is up. **(G,G′)** Scattergraphs of speed per macrophage per embryo over a 1-h period of random migration in controls and in the presence of *sSpitz*^*CS*^ **(G)** (*n* = 15, 17; *p* = 0.0229) or Spitz^sec^ **(G′)** (*n* = 15, 18; *p* = 0.858). **(H,H′)** Images of macrophages on the ventral midline at stage 15 in embryos containing GFP-tagged EGFR (EGFR-sfGFP) under the control of its endogenous promoter **(H)** and a control embryo lacking a modified *EGFR* locus **(H′)**. Embryos were immunostained for GFP (green in merge) and Fascin (magenta in merge) to reveal *EGFR-sfGFP* expression and identify macrophages, respectively; panels to the right of the merged images show contrast enhanced GFP channel. Scale bars denote 10 μm **(A,F)** and 5 μm **(H,H′)**; lines and error bars represent mean and standard deviation on scattergraphs, respectively; significance bars denote ^ns^*p* > 0.05, ^∗^*p* < 0.05, ^∗∗^*p* < 0.01, and ^****^p < 0.0001, respectively; statistical comparisons made via unpaired, two-tailed Student’s *t*-test **(C,E,E′,G,G′)** or Mann–Whitney test **(B,B′,C′,D,D′)**. Embryo genotypes are as follows: *w; Srp-GAL4,UAS-GFP/*+*; Crq-GAL4,UAS-GFP/*+(Control), *w; Srp-GAL4,UAS-GFP/UAS-sSpitz^CS^; Crq-GAL4,UAS-GFP/*+(sSpi^CS^), *w; Srp-GAL4,UAS-GFP/*+*; Crq-GAL4,UAS-GFP/UAS-Spitz^*Sec*^* (Spi^sec^), *w; EGFR-sfGFP* **(H)** and *w* **(H′)**.

Numbers of macrophages at the dorsal vessel were counted manually from maximum projections, with the total number in this field of view counted; recruitment to the dorsal vessel was defined as those macrophages contacting the dorsal vessel in a 100 μm long region corresponding to its medial-most section. The distance of macrophages from the dorsal vessel was measured using the points to line distance plugin in Fiji (macro made by Olivier Burri, EPFL, Lausanne). Similarly, developmental dispersal was quantified by counting numbers of macrophages on the ventral side of the embryo at stage 15 (Figures 1C,C′) or on the ventral midline (Supplementary Figures 1G,G′) in fields of view corresponding to the most-medial region of the embryo.

To quantify numbers of macrophages per embryo, maximum projections were assembled of lateral views comprising embryos imaged from their epithelial surface to the midline. Numbers of macrophages labeled with a nuclear marker (Red stinger) were counted manually using the point selection tool in Fiji and correspond to half the total number of macrophages per embryo.

To quantify macrophage wound responses, wound areas were first annotated from brightfield images taken at the 1-h timepoint. The number of macrophages in contact with and/or within the perimeter of the wound at 1-h post-injury were scored as having responded. The wound response is the number of responding macrophages divided by the wound area, normalized to the control average. The percentage of cells responding to wounds (% responders), a measure that allows normalization in case of varying numbers of macrophages in the wounding area, was calculated from those macrophages visible in the field of view pre-wounding that then migrated to the wound. To assess the range over which wound cues can be sensed, the shortest distance between the center of a non-responding macrophage in the pre-wound image and the wound edge (taken from the 60-min, post-wound brightfield image) was measured manually in Fiji and averaged per embryo.

### Statistical Analyses and Data Availability

Numerical data was collated in Microsoft Excel and statistical analyses performed in GraphPad Prism 9. Outliers were identified and removed from datasets using the Prism 9 ROUT method (where *Q* = 1). Prior to application of statistics for comparison between conditions, datasets were first analyzed using the suite of normality and logarithmic tests built into Prism 9. This program applies four different statistic tests to the chosen datasets (Anderson-Darling, D’Agostino and Pearson, Shapiro–Wilk and Kolmogorov–Smirnov tests). For all datasets comparing two conditions, a result of non-normality in any these tests (*p* < 0.05) led to us apply a non-parametric statistical test. Numerical data was then statistically analyzed using unpaired, two-tailed Student’s *t*-tests or Mann–Whitney tests to compare means for parametric and non-parametric data, respectively. Where greater than two means were compared, a one-way ANOVA with a Dunnett’s post-test was used. *P*-values were reported as significant at a threshold of *p* < 0.05. All manual data analysis was conducted on blinded datasets. Quoted n numbers in legends refer to the number of *Drosophila* embryos analyzed, with individual macrophage values used to calculate averages per macrophage, per embryo. Raw numerical data and images are available on request from the authors.

## Results

### Spitz Alters the Morphology and Migration Dynamics of *Drosophila* Embryonic Macrophages

Given the role of *Drosophila* EGFs in regulation of border cell migration in the oocyte and stem cell migration in the midgut (Duchek and Rørth, 2001; Liang et al., 2017), we hypothesized that Spitz may also regulate macrophage behavior in the developing *Drosophila* embryo. Since Spitz requires proteolytic cleavage for activation, two constituently-active variants of Spitz were used: Spitz^sec^ and sSpitz^CS^ (Ghiglione et al., 2002; Miura et al., 2006). In contrast to wild-type Spitz, these variants do not require cleavage via Rhomboid for their activation and secretion. Additionally, sSpitz^CS^ lacks a post-translational palmitoylation modification that normally restricts diffusion of wild-type ligand via interactions with plasma membranes (Miura et al., 2006). Comparison of these variants enables investigation of how the diffusion properties of Spitz contribute to alterations in macrophage behavior. Consequently, we expressed these Spitz variants specifically in macrophages to provide a local source of this growth factor, imaging GFP-labeled macrophages within developing embryos.

Our initial findings showed that developmental dispersal of macrophages was grossly normal on expression of either sSpitz^CS^ or Spitz^sec^ compared to controls at both stage 13 and stage 15/16 (Supplementary Figures 1A–F). Quantification of the numbers of macrophages on the ventral midline at stage 15 also showed no differences in the ability of macrophages to disperse over the embryo in the presence of either Spitz variant (Supplementary Figures 1G,G′). However, while macrophages were able to reach the ventral midline, the expression of Spitz appeared to alter their distribution, polarization and morphology in this region (Figure 1A). Analyzing the number of macrophages touching each other on the ventral midline showed that, while sSpitz^CS^-expressing macrophages had a wild-type distribution, Spitz^sec^ significantly increased the number of macrophages contacting one another, leading to the formation of cell clusters (Figures 1A–B′; asterisk in panel 1A).

It has previously been shown that overexpression of EGFR in larval blood cells (Zettervall et al., 2004) drives their overproliferation, presumably via autoactivation of this receptor tyrosine kinase. Similarly, removal of a negative regulator of EGFR signaling (Graf) also leads to expansion of larval blood cells (Kim et al., 2017). However, an increase in cell numbers cannot explain the clustering phenotype in the embryo (Figures 1A–B′), as we could not detect an increase in cell numbers on the ventral side of the embryo at stage 15 (Figures 1C,C′), nor was there an increase in overall numbers of macrophages in the embryo (Supplementary Figure 1H). This also suggests that, in contrast to the situation in larvae, EGFR signaling does not have the potential to drive macrophage proliferation in the embryo.

To analyze changes in macrophage morphology in more detail, macrophage polarization was assessed by measuring the aspect ratio (AR) of the cell body. In the presence of either sSpitz^CS^ or Spitz^sec^, macrophages were more elongated compared to controls lacking expression of either variant (Figures 1A,D,D′). Additionally, macrophages also appeared to contain fewer vacuoles in the presence of Spitz expression, structures previously established to contain engulfed apoptotic cells (Evans et al., 2013). Therefore, numbers of vacuoles can be used as an indirect read-out of macrophage efferocytosis. Quantification of the numbers of vacuoles per cell showed that in the presence of Spitz^sec^, but not sSpitz^CS^, macrophages contained fewer vacuoles and therefore were likely to contain fewer apoptotic cells (Figures 1E,E′). To assess if Spitz perturbed macrophage migration, macrophage movements (“random migration”) on the ventral midline of the embryo were tracked for 1 h at stage 15 (Figure 1F and Supplementary Movie 1). Expression of sSpitz^CS^ increased macrophage random migration speeds, but no difference was seen on expression of Spitz^sec^ (Figures 1G,G′). Taken together, these results show that macrophage-specific expression of active Spitz alters macrophage polarity, induces clustering and affects macrophage migration and phagocytosis in a variant-specific manner. The stimulation of macrophage polarization, clustering and increase in speed potentially indicates a role for Spitz as a macrophage chemoattractant, such as previously observed for border cells and gut stem cells in this organism (Duchek and Rørth, 2001; Liang et al., 2017). Alternatively, Spitz could operate as a chemokinetic molecule with a specific role in increasing migration speeds, though it is not clear how this might drive cluster formation. Consistent with the effects of activated Spitz on macrophage behavior and expression in larval blood cells (Kim et al., 2017), embryonic macrophages do indeed express EGFR (Figures 1H,H′), which can be visualized using a GFP-tagged version of this receptor (knocked into the endogenous *EGFR* locus; Revaitis et al., 2020). Furthermore, while there are high levels of activated ERK (DpERK) even within macrophages in control embryos (Supplementary Figure 2), the presence of either sSpitz^CS^ or Spitz^Sec^ enhances this read-out of EGFR activity within macrophages (Supplementary Figure 2).

### Cleavage Is Necessary for Spitz-Mediated Regulation of Macrophage Behavior

**FIGURE 2:**
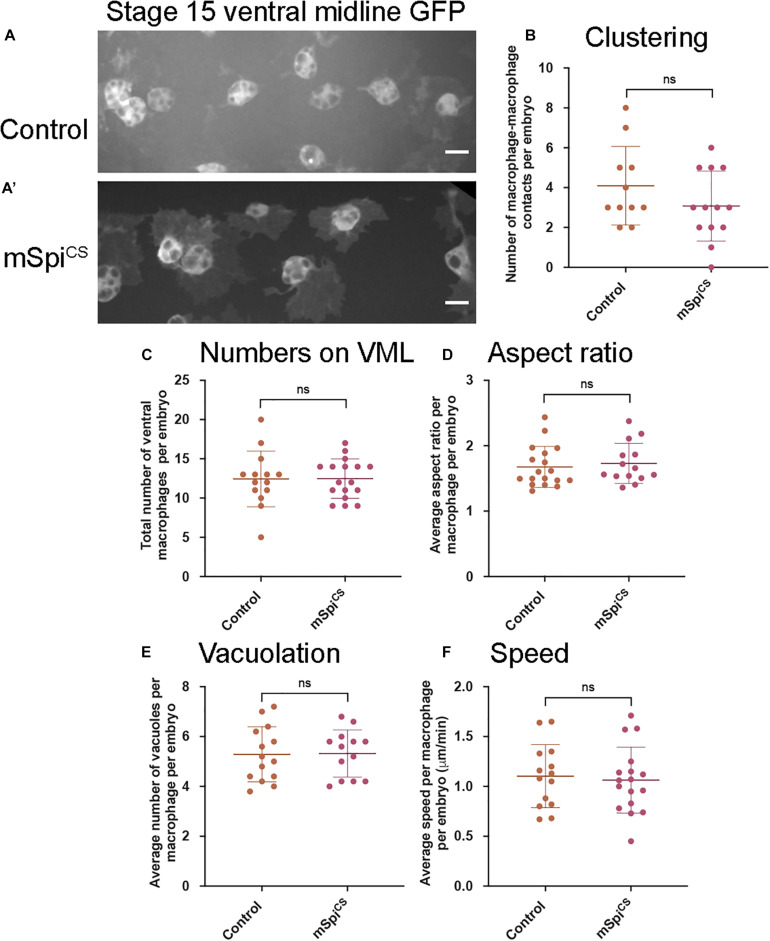
Expression of a membrane-bound form of Spitz fails to induce changes in embryonic macrophage behavior. **(A,A′)** Representative images of GFP-labeled macrophages in control embryos **(A)** and embryos containing macrophages expressing mSpi^CS^-GFP **(A′)** on the ventral midline at stage 15; mSpi^CS^-GFP macrophages appear more defined due to additional GFP expression due to the GFP tag that is part of the mSpi^CS^-GFP transgene; scale bars represent 10 μm. **(B–F)** Scattergraphs showing number of macrophage-macrophage contacts per embryo to assay macrophage clustering **(B)** (*n* = 11, 13; *p* = 0.690), numbers of macrophages on the ventral midline **(C)** (*n* = 14, 17; *p* = 0.188), cell body aspect ratio per macrophage, per embryo **(D)** (*n* = 18, 14; *p* = 0.464), vacuoles per macrophage, per embryo **(E)** (*n* = 14, 13; *p* = 0.926) and random migration speed in μm per minute **(F)** (*n* = 14, 17; *p* = 0.743) at stage 15 in controls and embryos with macrophage-specific expression of mSpitz^CS^. Lines and error bars represent mean and standard deviation on scattergraphs, respectively; significance bars denote *p* > 0.05 (ns); statistical comparisons made via Mann–Whitney test **(D)** or unpaired, two-tailed Student’s *t*-test **(B,C,E,F)**. Embryo genotypes are as follows: *w; Srp-GAL4,UAS-GFP/*+*; Crq-GAL4,UAS-GFP/*+(Control), *w; Srp-GAL4,UAS-GFP/*+*; Crq-GAL4,UAS-GFP/UAS-mSpitz^CS^-GFP* (mSpi^CS^).

To investigate whether release from the membrane is required for Spitz-induced changes in macrophage behavior, a membrane-bound variant (mSpitz^CS^; Miura et al., 2006) was expressed. Expression of mSpitz^CS^ did not alter macrophage clustering, numbers of cells in the ventral region, their morphology, vacuolation or migration speeds on the ventral midline (Figure 2). This suggests that cleavage and release of Spitz from the plasma membrane is needed for induction of macrophage phenotypes and that these phenotypes are not a non-specific consequence of overexpression of Spitz.

### Tissue-Specific Release of Spitz Alters Macrophage Localization and Vacuolation

**FIGURE 3:**
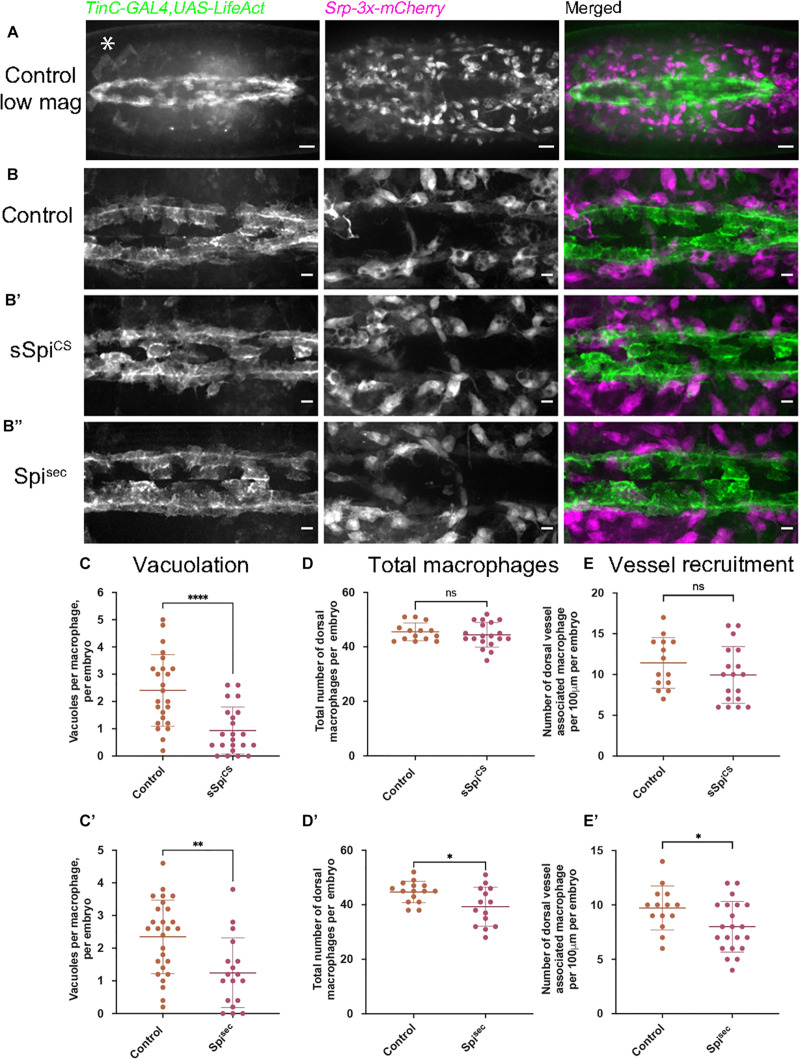
Expression of Spitz in the developing fly heart alters macrophage localization and vacuolation. **(A–B′′)** Maximum projections of dorsal side of stage 15 embryos showing the dorsal vessel (labeled using *TinC-GAL4,UAS-LifeAct*; green in merge) and associated macrophages (labeled via *Srp-3x-mCherry*) in controls **(A,B)** and embryos with *TinC-GAL4* mediated expression of *sSpitz*^*CS*^ **(B′)** or *Spitz*^*Sec*^ **(B′′)**. Anterior is right; asterisk (^∗^) denotes *TinC-GAL4* driven expression in lateral regions away from the dorsal vessel. **(C,C′)** Scattergraphs of vacuole counts per macrophage in controls and in the presence of dorsal vessel-expressed sSpitz^CS^ **(C)** (*n* = 24, 22; *p* < 0.0001) or Spitz^sec^ **(C′)** (*n* = 27, 18; *p* = 0.0020). **(D,D′)** Scattergraphs of the total number of macrophages present in the field of view at the dorsal face in controls and in the presence of dorsal vessel-expressed sSpitz^CS^ **(D)** (*n* = 14, 19; *p* = 0.497) or Spitz^sec^ **(D′)** (*n* = 15, 14; *p* = 0.0170). **(E,E′)** Scattergraphs of the number of macrophages contacting the dorsal vessel in controls and in the presence of dorsal vessel-expressed sSpitz^CS^ **(E)** (*n* = 14, 18; *p* = 0.221) or Spitz^sec^ **(E′)** (*n* = 14, 21; *p* = 0.0312). Scale bars denote 20 μm **(A)** and 10 μm **(B–B′′)**; lines and error bars represent mean and standard deviation on scattergraphs, respectively; significance bars denote ^ns^*p* > 0.05, ^∗^*p* < 0.05, ^∗∗^*p* < 0.01, ^****^p < 0.0001, respectively; statistical comparisons made via a Mann–Whitney test **(C,D)** or unpaired, two-tailed Student’s *t*-test **(C′,D′,E,E′)**. Embryo genotypes are as follows: *w; TinC-GAL4,UAS-LifeAct,Srp-3x-mCherry/*+(Control)*, w;*+*/UAS-sSpitz^*CS*^; TinC-GAL4,UAS-LifeAct,Srp-3x-mCherry/*+(sSpi^CS^) and *w; TinC-GAL4,UAS-LifeAct,Srp-3x-mCherry/UAS-Spitz^*sec*^* (Spi^sec^).

Given that under normal conditions, macrophages may not be the source of activated Spitz within the embryo, and also to avoid longer-term expression of Spitz by these cells, Spitz was expressed in an independent tissue that macrophages encounter during their dispersal. Thus, sSpitz^CS^ or Spitz^sec^ were expressed in the developing heart, a structure called the dorsal vessel, using *TinC-GAL4*, a driver derived from the enhancer region of *Tinman* (Lo and Frasch, 2001). *Tinman* encodes a transcription factor expressed across the early embryonic mesoderm before becoming restricted to the progenitor heart and lateral visceral muscles by stage 15 (Bodmer, 1993). During development, clusters of cardiocytes begin to form the dorsal vessel, which is then colonized by migrating macrophages (Figure 3A). We hypothesized that misexpression of Spitz in the dorsal vessel would alter macrophage morphology and behavior, enabling us to determine whether cell-autonomous expression was necessary for the effects of Spitz expression and confirm our previous results using macrophage-specific expression.

Embryos with LifeAct-labeled cardiocytes expressing either sSpitz^CS^ or Spitz^sec^ were mounted dorsally and imaged at the most-medial point of the developing dorsal vessel and compared to controls lacking Spitz expression (Figures 3B–B′′); macrophages were labeled using the GAL4-independent *Srp-3x-mCherry* reporter construct (Gyoergy et al., 2018). The presence of either sSpitz^CS^ or Spitz^sec^ appeared to inhibit phagocytic uptake of apoptotic cells, since dorsal vessel-associated macrophages contained significantly fewer vacuoles compared to controls (Figures 3C,C′), consistent with phenotypes achieved using macrophage-specific expression of Spitz. Quantification of macrophage dispersal showed that expression of Spitz^sec^, but not sSpitz^CS^, marginally decreased the total numbers of macrophages recruited to this dorsal region (Figures 3D,D′). Similarly, only Spitz^sec^ altered the precise localization of dorsal vessel-associated macrophages, with fewer directly associated with the dorsal vessel itself (Figures 3E,E′). The reduction in macrophages at the dorsal vessel in the presence of Spitz^sec^ would appear counterintuitive to the hypothesis that Spitz may operate as a macrophage chemoattractant, however, *TinC-GAL4* also drives expression in regions lateral to the dorsal vessel (Azpiazu and Frasch, 1993; e.g., asterisk in Figure 3A) and this may be responsible for recruitment of macrophages away from the dorsal vessel. The lack of a stronger phenotype may indicate that Spitz can only act over short ranges. Interestingly, the observed phenotypes corroborate the potential decrease in apoptotic clearance by macrophages in the presence of Spitz. However, in this instance both variants of Spitz were competent to induce this phenotype. The consistent reduction of efferocytosis in macrophages exposed to Spitz^sec^ at the ventral midline and dorsal vessel led us to examine how Spitz affects apoptotic cell-macrophage interactions in more detail.

### Exposure to Spitz Reduces the Efferocytic Capacity of Macrophages

**FIGURE 4:**
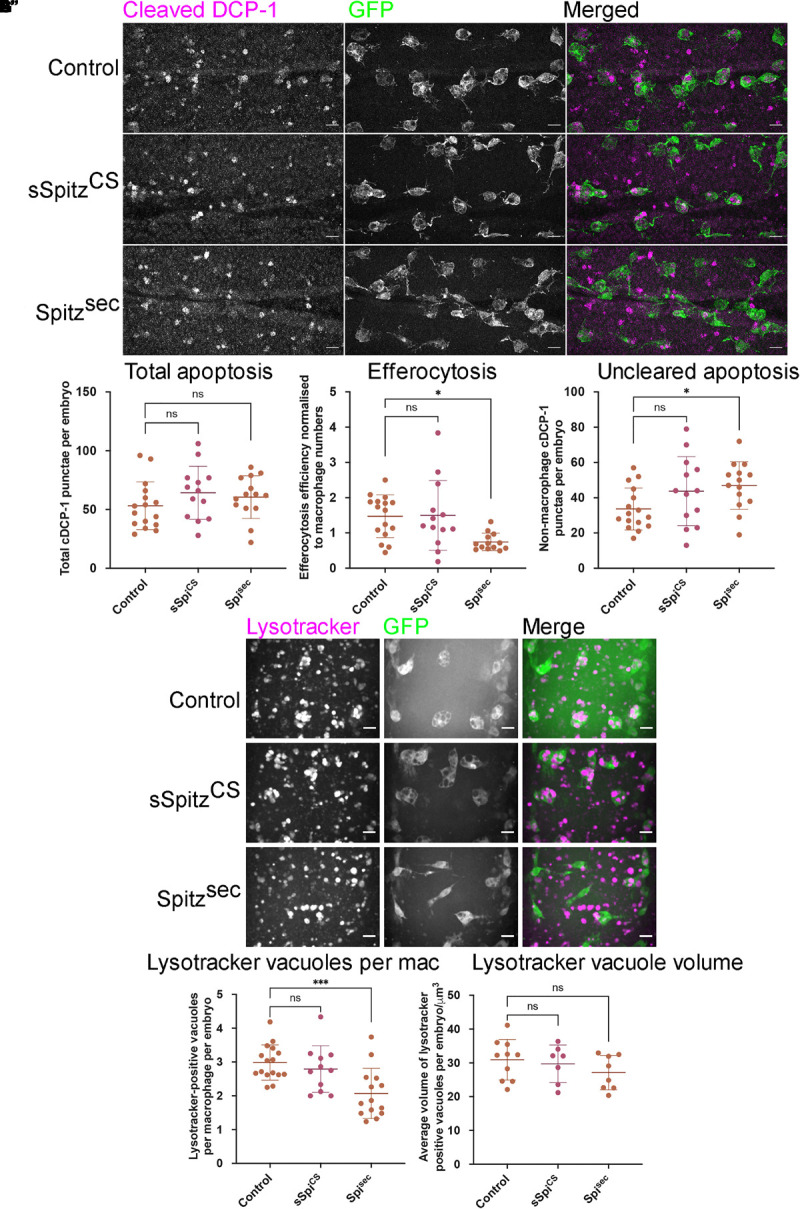
Spitz impairs macrophage-mediated apoptotic cell clearance. **(A)** Maximum projections of the ventral region of stage 15 embryos immunostained for cleaved DCP-1 (cDCP-1; magenta in merge) and GFP (green in merge). Control embryos **(A)** were compared with embryos in which sSpitz^CS^ **(A′)** or *Spitz*^*Se*c^ **(A′′)** were specifically expressed in macrophages. **(B)** Scattergraph showing the total numbers of cDCP-1 positive punctae present within the ventral field of view per embryo **(B)** (*n* = 16, 13, 14; *p* = 0.265 and *p* = 0.519 for comparison of control vs. sSpi^CS^ and control vs. Spi^sec^, respectively). **(C)** Scattergraph showing efficiency of apoptotic cell clearance/efferocytosis (percentage of cDCP-1 punctae engulfed by macrophages per field of view, per embryo, normalized according to numbers of macrophages in the field of view; *n* = 16, 13, 13; *p* = 0.994 and *p* = 0.0125 for comparison of control vs. sSpi^CS^ and control vs. Spi^sec^, respectively). **(D)** Scattergraph showing average number of cDCP-1 punctae not engulfed by macrophages per field of view, per embryo (*n* = 16, 13, 14; *p* = 0.142 and *p* = 0.0386 for comparison of control vs. sSpi^CS^ and control vs. Spi^sec^, respectively). **(E–E′′)** Images of macrophages (green in merge) with acidified phagosomes labeled using lysotracker red (magenta in merge) at stage 15 on the ventral midline in genotypes indicated. **(F)** Scattergraph showing numbers of lysotracker-positive punctae per macrophage, per embryo in the presence and absence of Spitz expression (*n* = 16, 11, 14; *p* = 0.678 and *p* = 0.0009 for comparison of control vs. sSpi^CS^ and control vs. Spi^sec^, respectively). **(G)** Scattergraph showing average volume of lysotracker-positive phagosomes per macrophage, per embryo in the presence and absence of Spitz expression (*n* = 10, 7, 8; *p* = 0.873 and *p* = 0.284 for comparison of control vs. sSpi^CS^ and control vs. Spi^sec^, respectively). Scale bars denote 10μm; lines and error bars represent mean and standard deviation on scattergraphs, respectively; significance bars denote ^ns^*p* > 0.05, ^∗^*p* < 0.05, and ^∗∗∗^*p* < 0.001, respectively; all statistical comparisons made via one-way ANOVA with a Dunnett’s post-test. Embryo genotypes are as follows: *w; Srp-GAL4,UAS-GFP/*+*; Crq-GAL4,UAS-GFP/*+ (Control), *w; Srp-GAL4,UAS-GFP/UAS-sSpitz^CS^; Crq-GAL4,UAS-GFP/*+ (sSpi^CS^) and *w;Srp-GAL4,UAS-GFP/*+*; Crq-GAL4,UAS-GFP/UAS-Spitz^*Sec*^* (Spi^sec^).

Expression of Spitz in macrophages or the dorsal vessel induced a loss of vacuoles assumed to contain apoptotic cells within macrophages on the ventral midline or dorsal surface, respectively, suggesting that Spitz can interfere with apoptotic cell clearance (efferocytosis). It is also possible that expression of this growth factor alters overall levels of apoptosis in the developing embryo, such that there are fewer corpses for macrophages to clear. Therefore, to address the effects of Spitz on apoptotic cell clearance, embryos with or without macrophage-specific expression of either sSpitz^CS^ or Spitz^sec^ were immunostained for the cleaved form of the *Drosophila* caspase DCP-1 (cDCP-1), which can be used as a proxy for apoptotic cells (Song et al., 1997; Figures 4A–A′′). To quantify the efficiency of efferocytosis, we counted the total number of cDCP-1 punctae on the ventral side of the embryo at stage 15 and calculated the proportion of these engulfed by macrophages. Macrophage-specific expression of either Spitz variant did not alter the total numbers of apoptotic cells in these regions compared to control embryos (Figures 4A–B). This suggests that Spitz does not inhibit apoptosis of surrounding cells, nor does it cause a dramatic build-up of apoptotic corpses due to the reduction in engulfment by macrophages. As per the analysis of macrophage vacuolation (Figures 1E,E′), there was a decrease in the relative efficiency of apoptotic cell clearance specific to the expression of Spitz^sec^, with a lower proportion of cDCP-1 punctae present within macrophages in this genotype (Figure 4C). This decrease in apoptotic cell clearance led to a small but significant increase in the number of cDCP-1 punctae outside of macrophages (Figure 4D).

To check that the decrease in vacuoles and cDCP-1 punctae was not a consequence of more rapid phagosome maturation, acidification of phagosomes was investigated using lysotracker staining (Figures 4E–E″). As per cDCP-1 staining, there was a significant decrease in the number of acidified phagosomes in the presence of Spitz^sec^ but not sSpitz^CS^, compared to controls (Figures 4E–F). Importantly, there was no difference in the sizes of lysotracker-positive phagosomes between experimental conditions (Figure 4G), suggesting it is not the case that phagosomes mature and fuse at a faster rate in the presence of Spitz.

These data therefore support the idea that less apoptotic cell clearance is being carried out by macrophages in the presence of Spitz, but without the consequence of large changes in the number of cells undergoing cell death or remaining uncleared by phagocytes. That these phenotypes were again specific to Spitz^sec^ reinforces the idea that differences between these two Spitz variants may prevent sSpitz^CS^ from acting locally in some contexts. Having established that Spitz^sec^ decreases efficiency of macrophage-mediated efferocytosis in addition to its impact on cell morphology, we sought to establish if Spitz was able to disrupt macrophage chemotaxis to non-developmental stimuli.

### Exposure to Spitz Dampens WoundResponses in Macrophages in a Distance-Dependant Manner

**FIGURE 5:**
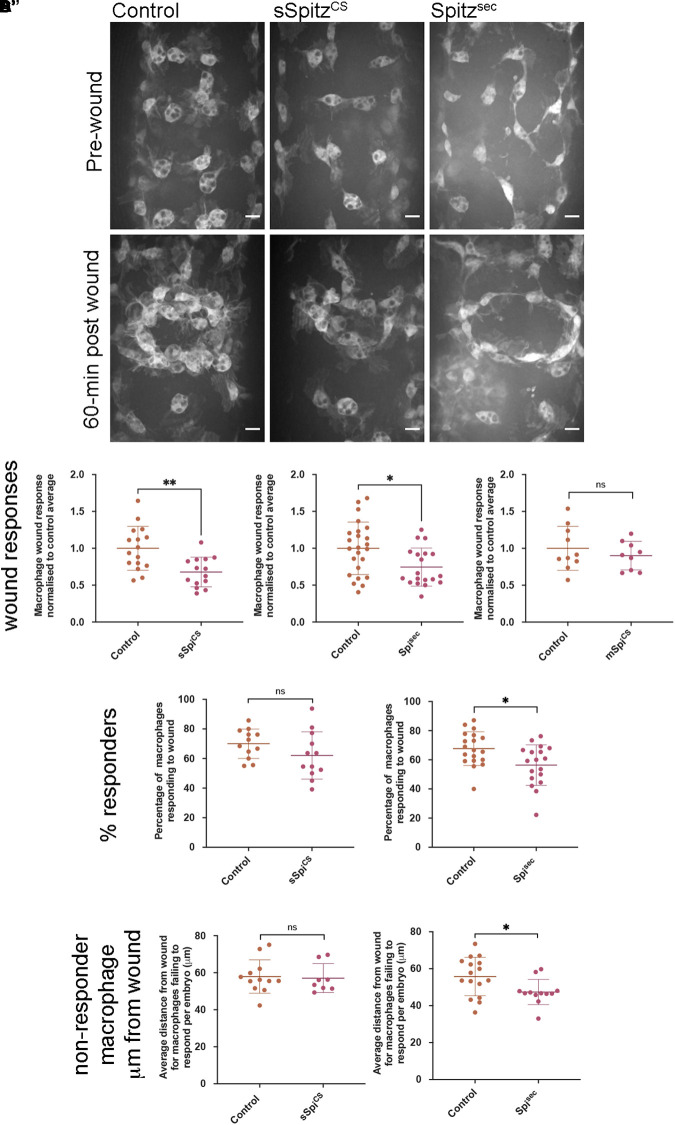
Spitz impairs macrophage wound responses. **(A)** Maximum projections showing images of GFP-labeled macrophages on the ventral midline region in controls and in embryos containing macrophages expressing sSpitz^CS^ or Spitz^sec^; upper panels show stage 15 embryos immediately prior to wounding; lower panels show corresponding embryo 1-h post wounding. **(B–B′′)** Scattergraphs showing macrophage wound responses (number of macrophages responding to the wound normalized to wound area and to control responses) in controls and embryos with macrophage-specific expression of sSpitz^CS^ **(B)** (*n* = 16, 14; *p* = 0.0020), Spitz^sec^ **(B′)** (*n* = 24, 19; *p* = 0.0160), or mSpitz^CS^ (*n* = 9, 10; *p* = 0.406). **(C,C′)** Scattergraphs showing percentage of macrophages responding to wound per embryo (% of those in the field of view that reach the wound) per embryo for control embryos compared to embryos with macrophage specific-expression of sSpitz^CS^ **(C)** (*n* = 12, 12; *p* = 0.159) or Spitz^sec^ **(C′)** (*n* = 19, 18; *p* = 0.0105). **(D,D′)** Scattergraphs showing average distance from the wound edge (immediately prior to wounding) of those macrophages that fail to respond, per embryo, for control embryos compared to embryos with macrophage specific-expression of sSpitz^CS^ **(D)** (*n* = 12, 8; *p* = 0.678) or Spitz^sec^ **(D′)** (*n* = 16, 12; *p* = 0.0421). Scale bars denote 10 μm; lines and error bars represent mean and standard deviation on scattergraphs, respectively; significance bars denote ^*ns*^*p* > 0.05, ^∗^*p* < 0.05, and ^∗∗^*p* < 0.01, respectively; statistical comparisons made via a Mann–Whitney test **(B′,B′′,D,D′)** or an unpaired, two-tailed Student’s *t*-test **(B,C,C′)**. Embryo genotypes are as follows: *w; Srp-GAL4,UAS-GFP/*+*; Crq-GAL4,UAS-GFP/*+ **(Control)**, *w; Srp-GAL4,UAS-GFP/UAS-sSpitz^CS^; Crq-GAL4,UAS-GFP/*+ (sSpi^CS^), *w;Srp-GAL4,UAS-GFP/*+*; Crq-GAL4,UAS-GFP/UAS-Spitz^*Sec*^* (Spi^sec^) and *w; Srp-GAL4,UAS-GFP/*+*; Crq-GAL4,UAS-GFP/UAS-mSpitz^CS^-GFP* (mSpi^CS^).

*Drosophila* embryonic macrophages exhibit robust wound responses by polarizing toward and then migrating to sites of tissue damage (Stramer et al., 2005). These cells are refractile to wounding stimuli prior to late stage 14 due to persisting developmental signals, although they are still able to chemotax toward and engulf cells undergoing apoptotic cell death at this point in development (Moreira et al., 2010). This suggests that a hierarchy between different signals and that the integration of those signals can impact wound responses. Therefore, to address the effects of Spitz on inflammatory responses to injury, controls and embryos containing macrophage-specific expression of sSpitz^CS^ or Spitz^sec^ were laser wounded on the ventral surface of the embryo at stage 15 and the subsequent responses of GFP-labeled macrophages imaged (Figure 5A and Supplementary Movie 2). One-hour post wounding, there was a significant reduction in the macrophage wound response (number of macrophages at the wound divided by wound area, normalized to the control average) in the presence of either Spitz^sec^ or sSpitz^CS^ compared to controls (Figures 5A–B′). As previously, cleavage of Spitz appears necessary to induce changes in macrophage behavior, since expression of a membrane-bound form of Spitz (mSpitz^CS^) failed to impact macrophage recruitment to wounds (Figure 5B′′). The decrease in wound responses was paralleled by a decrease in the percentage of cells present in the field of view prior to wounding that are able to respond to injury for Spitz^sec^ but not sSpitz^CS^ (Figures 5C,C′), again highlighting the stronger effect of this variant.

Given the lack of a dramatic change in the numbers of apoptotic cells in the embryonic environment on expression of Spitz (Figures 4A–B), it seems unlikely that distraction of macrophages by apoptotic cells (e.g., as observed in Roddie et al., 2019) accounts for this phenotype. Instead, a loss of sensitivity to wound signals at more distal sites, where wound cues may be weaker, could explain why a smaller proportion of macrophages respond. Therefore, to assess whether the loss of responses from regions further away from the wound site explained the reduction in numbers of macrophages reaching wounds, the distances of non-responsive macrophages from wound edges was measured. There were no differences in these measurements when sSpitz^CS^ and control embryos were compared (Figure 5D), however, the average distances of non-responding macrophages from the wound were significantly lower in the presence of Spitz^sec^ compared to controls (Figure 5D′). This shows that macrophages further away from the wound site are less likely to respond in the presence of Spitz^sec^. Thus, a loss of recruitment of more distal macrophages to wound sites in the presence of Spitz^sec^ contributes to impaired wound responses. Potentially, those cells absent from wound sites in the presence of sSpitz^CS^ are those that would otherwise migrate from regions outside of the field of views used for this particular analysis; this also potentially explains the lack of a difference in the percentage of cells that respond in the pre-wound field of view (Figure 5C).

In conclusion, the impairment of macrophage inflammatory responses in the presence of either Spitz^sec^ or sSpitz^CS^ highlights the capacity of this molecule to regulate a range of innate immune behaviors that depend on efficient polarization and migration in the developing *Drosophila* embryo (see Table 1 for a summary of phenotypes). The fact that Spitz specifically impacts the recruitment of more distal macrophages to wound sites would indicate it modulates or overrides the ability of these important cells to sense or respond to those signals produced at wound sites or by apoptotic cells.

**TABLE 1:**
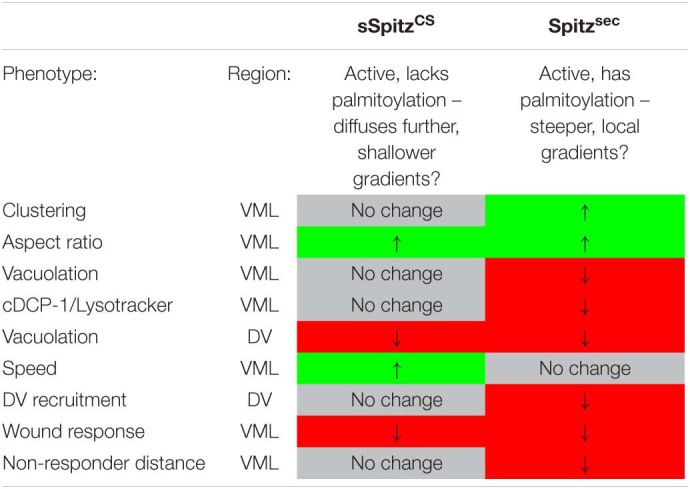
Summary of Spitz-induced macrophage phenotypes.

*VML, ventral midline; DV, dorsal vessel. mSpi^CS^ does not induce any macrophage phenotypes.*

## Discussion

Here we show for the first time that the *Drosophila* epidermal growth factor pathway modifies immune cell function in the developing embryo, representing a new cue regulating the behavior of this organism’s macrophages *in vivo.* De-regulated release of Spitz disrupts macrophage migration, induces elongation and perturbs the ability of macrophages to respond to wounds and clear apoptotic cells. In this study, two variants of protease-independent Spitz with different diffusion properties were used to assess the role of Spitz as a macrophage chemoattractant in the *Drosophila* embryo: macrophage phenotypes varied according to the activated variant of Spitz used, suggesting different physical properties of this cue may influence macrophage behavior in subtle ways.

Two non-mutually exclusive scenarios may explain how Spitz alters macrophage morphology and speed: regulation of macrophage motility by Spitz, which causes an override of endogenous signals, and/or reprogramming of macrophages to different activation states with different migratory and morphological characteristics. EGF ligands can function as chemoattractants in a number of situations: human EGF and the *Drosophila* EGF ligand Gurken regulate monocyte chemotaxis and *Drosophila* border cell migration, respectively (Duchek and Rørth, 2001; Lamb et al., 2004), while Spitz was itself identified as a stem cell attractant released by *Drosophila* midgut cells undergoing apoptosis (Liang et al., 2017). We found that, in the presence of Spitz, macrophages on the ventral midline became more highly polarized and migrated at greater speeds (sSpitz^CS^ specifically). The changes in cell shape could reflect changes in their migratory abilities, or be indicative of a chemotactic response toward a gradient (Sarris and Sixt, 2015), through enhanced formation or stabilization of a cell’s leading edge. Alternatively, they may be the result of macrophage reprogramming events that have previously been linked to morphological distinctions between pro-inflammatory and anti-inflammatory macrophages (McWhorter et al., 2013). Single cell RNA sequencing studies have shown that blood cell populations may be more complicated in *Drosophila* than previously anticipated (Cattenoz et al., 2020; Cho et al., 2020; Tattikota et al., 2020). At present these approaches are limited to larval stages, although recent work suggests that subpopulations of functionally-distinct macrophages may also exist in the developing fly embryo (Coates et al., 2020). The expression of Spitz under the control of *TinC-GAL4* corroborated midline efferocytosis defects and revealed that macrophage-specific expression was not necessary for an impact on this behavior. The reduction in the numbers of macrophages at the dorsal vessel on expression of Spitz^sec^ is possibly the result of distal *TinC-GAL4* activity within the lateral visceral muscles (Bodmer, 1993) attracting macrophages away from this tissue. Again, the fact that Spitz^sec^, but not sSpitz^CS^ altered macrophage recruitment in this region potentially reflects the stronger phenotypes obtained with Spitz^sec^, which may in turn relate to the differences in diffusion of these two variants (see below).

EGF signaling can block apoptosis through its action as pro-survival signal (Henson and Gibson, 2006) and can also stimulate compensatory proliferation (Fogarty and Bergmann, 2017). However, Spitz expression did not alter the total number of apoptotic cells *in vivo* but did impair clearance by macrophages. While macrophages express EGFR and downstream signaling pathways are activated via exposure to either Spitz variant, we cannot exclude the possibility that EGFR signaling in cells other than macrophages contributes to the phenotypes we describe, since EGFR is widely expressed in the developing embryo (Revaitis et al., 2020). Spitz-induced phenotypes may reflect reprogramming to an activation state that is less efficient at engulfing dying cells, since the capacity to clear apoptotic cells can vary across macrophage subpopulations in some organisms (Zizzo et al., 2012). Unrestrained EGFR signaling has previously been demonstrated to drive proliferation of larval blood cells (Zettervall et al., 2004; Kim et al., 2017), but has also been implicated in acquisition of a lamellocyte fate in the presence of elevated reactive oxygen species (Sinenko et al., 2012), pointing toward the potential for a role in the alteration of cell specification in *Drosophila* blood lineages.

Alternatively, chemotaxis or chemokinesis regulated by Spitz or signal integration mechanisms may impair detection of apoptotic corpses – if Spitz represents a chemoattractant or reprogramming factor it may distract macrophages from their clearance duties (e.g., toward each other leading to clustering or increased migration speeds). Intriguingly, Spitz and human EGF share similar processing and secretion mechanisms to a known find-me cue for apoptotic cells, Fractalkine (Sokolowski et al., 2014). Both Fractalkine and EGFs require activation via caspase-regulated proteases – Rhomboid and ADAM-17, respectively (Rose-John, 2013; Liang et al., 2017). The changes in macrophage shape and their responses to stimuli that are induced by Spitz, and the similarities in how Spitz and other find-me cues are secreted, raises the potential role of Spitz as a chemoattractant used in the “find-me” phase of efferocytosis (Ravichandran, 2003). High levels of this cue may therefore interfere with detection of apoptotic cells (i.e., signals from apoptotic cells are “drowned out” by misexpressed Spitz), though considerably more work would be required to establish Spitz as a find-me cue. Furthermore, these experiments are not straightforward given the role of EGF signaling in midline development (Golembo et al., 1996) and the fact that disruption of that process blocks macrophage dispersal (Paladi and Tepass, 2004; Evans et al., 2010a).

Similarly, we have shown that the presence of either Spitz^sec^ or sSpitz^CS^, inhibits the ability of macrophages to respond to wounding stimuli. Uncleared apoptotic cells can impair wound responses in the developing embryo (Roddie et al., 2019). However, there was only a mild increase in the number of uncleared apoptotic cells, potentially as glial cells and epidermal cells may compensate for the decreases in macrophage-mediated clearance. Since no substantial changes in overall levels of apoptosis were detected in the presence of either Spitz variant, nor did sSpitz^CS^ expression impact efferocytosis on the ventral midline yet still altered wound responses, we do not favor the explanation that uncleared apoptotic cells undermine macrophage inflammatory responses to injury in this context. Instead impairment of wound responses may result from competition between Spitz and the damage-associated molecular signals released at wound sites (Koh and DiPietro, 2011), or relate to macrophage reprogramming as discussed above. Indeed, our results showed that Spitz prevented more distal macrophages from responding to wounds, supporting the idea of competing chemotactic gradients as opposed to a general reprogramming of macrophages that results in desensitization to wound signals. This disruption of macrophage responses may be specific to regions of the embryo more distal to wound sites, as wound signals may be present at lower concentrations in those environments.

The phenotypic differences observed between Spitz^sec^ and sSpitz^CS^ likely reflect the absence of a palmitoyl group in the latter (Miura et al., 2006). However, we have been unable to confirm equivalent levels of expression of these variants (data not shown), as it is not clear that the anti-Spitz antibodies we have at our disposal recognize the sSpitz^CS^ variant, which contains a mutation in the region used to generate that antibody (Schweitzer et al., 1995). Therefore, it remains possible that differences in expression levels of these variants contribute to differences in the phenotypes we have observed. Palmitoylation is known to increase tethering of ligands at the plasma membrane post-secretion (Salaun et al., 2010). Additionally, mutation in the palmitoylation site of signaling proteins is known to alter cell–cell signaling, e.g., Fas-mediated cell death (Guardiola-Serrano et al., 2010), and significantly reduce diffusion speed of ligands (Sowa et al., 1999). This may allow sSpitz^CS^ to diffuse further from its source, forming shallower gradients over longer distances that are more difficult for cells to interpret. In contrast, Spitz^sec^ would remain more highly concentrated at its source leading to steeper gradients over a shorter range. This potentially explains why Spitz^sec^ can drive macrophage clustering between neighboring macrophages. Shallower, more long-range gradients may enable increased migration speeds with sSpitz^CS^, whereas Spitz^sec^ promotes clustering more locally. Given that membrane-bound Spitz does not drive clustering, this suggests cluster formation is not the result of cell–cell adhesion via receptor-ligand pairing.

Expression of variants at the dorsal vessel acts as a more defined point source of Spitz and this may explain why sSpitz^CS^ is more effective here than when expressed in macrophages, although differences in expression or stability of these different variants may also contribute. The lack of recruitment to the dorsal vessel may be due to recruitment to areas of *TinC-GAL4* expression elsewhere in the embryo. Expression of the PDGF/Vegf-related ligand Pvf2 is sufficient to retain macrophages in the head of the embryo (Evans et al., 2010b), therefore this molecule is capable of exerting a more profound effect than Spitz on macrophages as they disperse. Therefore, in comparison to Pvf2, Spitz may exert weaker effects, act over a shorter range and/or merely stimulate migration speeds rather than function as a chemoattractant (i.e., function as a chemokinetic molecule).

Taken together, our results show that Spitz can alter macrophage migration and functional responses to wounds and apoptotic cells during *Drosophila* development. These processes are important immune cell behaviors that can become dysregulated in a diverse array of human conditions including cancer, atherosclerosis and chronic inflammatory conditions. These results therefore have clear implications for our understanding of the role that EGF ligands play during development and in the progression of chronic inflammation. Furthermore, these findings have implications and relevance to therapeutic strategies that seek to interfere with EGF signaling – indeed, targeting the EGF pathway shows promise as a therapeutic strategy in models of chronic inflammation (Qu et al., 2012; Omachi et al., 2017; Rahman et al., 2019). Future work will establish the exact mechanisms of actions via which *Drosophila* EGFs regulate macrophage function, the downstream signaling pathways involved and whether these functions are conserved through evolution.

## Data Availability Statement

The original contributions generated for this study are included in the article/Supplementary Material, further inquiries can be directed to the corresponding author.

## Author Contributions

All authors contributed to conception of the project, experimental design, and the preparation and editing of the manuscript. Experiments were conducted by OT, EA, and IE. LP and IE supervised and obtained funding for the project.

## Conflict of Interest

The authors declare that the research was conducted in the absence of any commercial or financial relationships that could be construed as a potential conflict of interest.
