# Supplementary figures and images for: The Epidermal Growth Factor Ligand Spitz Modulates Macrophage Efferocytosis, Wound Responses and Migration Dynamics During Drosophila Embryogenesis
Source: Front Cell Dev Biol. 2021 Apr 8;9:636024. doi: 10.3389/fcell.2021.636024 (PMC8060507; doi:10.3389/fcell.2021.636024)

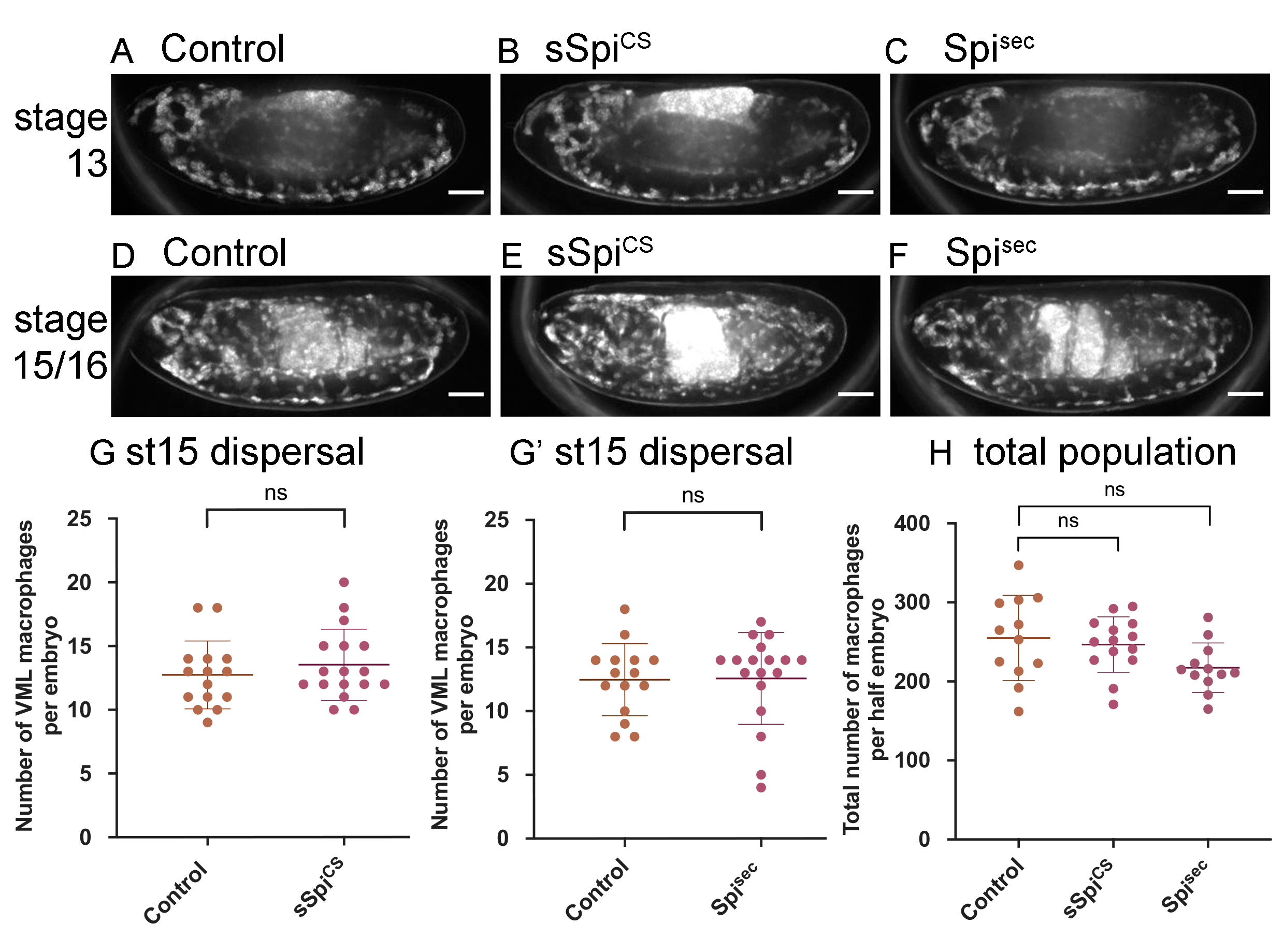

Supplement: Supplementary file 1 [file Image_1.TIF]

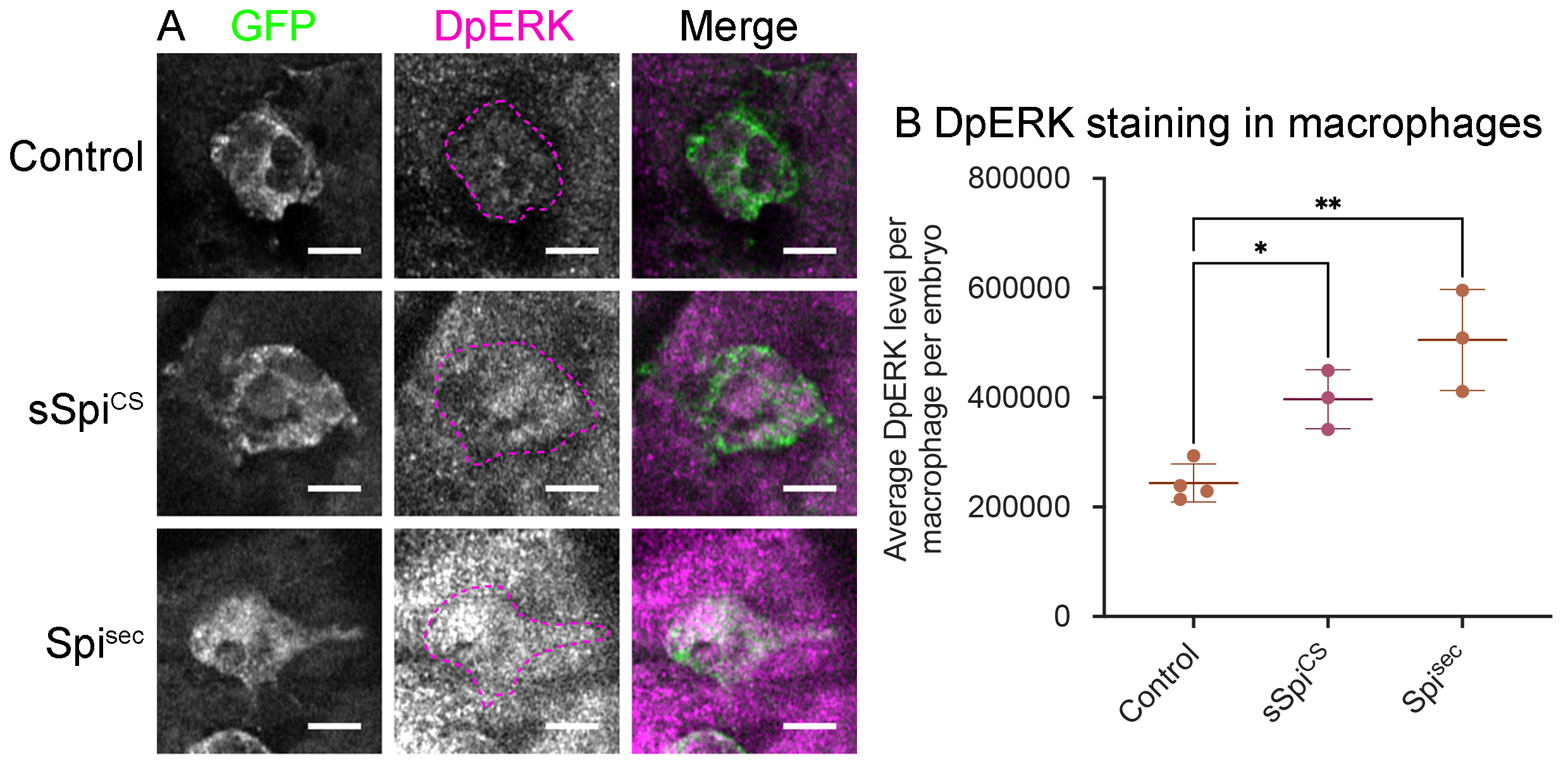

Supplement: Supplementary file 2 [file Image_2.TIF]
