# Supplementary material for: The Epidermal Growth Factor Ligand Spitz Modulates Macrophage Efferocytosis, Wound Responses and Migration Dynamics During Drosophila Embryogenesis
Source: Front Cell Dev Biol. 2021 Apr 8;9:636024. doi: 10.3389/fcell.2021.636024 (PMC8060507; doi:10.3389/fcell.2021.636024)
Supplement: Supplementary file 6 [file Data_Sheet_1.docx]

# Supplementary information – Tardy et al., 2021

## Supplementary Tables

**Supplementary Table 1. Experimental genotypes**

Table shows genotypes used in each figure.

## Supplementary Figure Legends

**Supplementary Figure 1. Macrophage-specific expression of Spitz does not alter developmental dispersal or total numbers of macrophages in the embryo**

**(A-F)** Lateral images showing developmental dispersal of macrophages at stage 13 **(A-C)** and stage 15/16 **(D-F)** in controls **(A, D)** and in the presence of macrophage-specific expression of sSpi^CS^ **(B, D)** or Spi^sec^ **(C, E)**. **(G-G’)** Scattergraphs showing number of macrophages per embryo on the ventral midline at stage 15 in controls and in the presence of macrophage-specific expression of sSpi^CS^ (n= 15, 17 embryos; *p* = 0.417 via an unpaired, two-tailed Student’s t-test) **(G)** or Spi^sec^ (n= 15, 18 embryos; *p* = 0.939 via an unpaired, two-tailed Student’s t-test) **(G')**. **(H)** Scattergraph showing number of macrophages per embryo at stage 15 in the indicated genotypes. Number of red stinger (nuclear RFP) labelled macrophages scored per half of an embryo – imaged laterally from most superficial side to midline (n = 12 control, 14 sSpi^CS^ and 12 Spi^sec^ embryos; one-way ANOVA with a Dunnett’s post-test used to compare control group with Spitz embryos; control vs. sSpi^CS^ *p* = 0.82, control vs. Spi^sec^ *p* = 0.056)**.** Scale bars denote 50 μm **(A-F)**; ns denotes not significant; lines and error bars represent mean and standard deviation, respectively. Embryo genotypes are as follows: *w;Srp-GAL4,UAS-GFP/+;Crq-GAL4,UAS-GFP/+* **(control; A, D, G-G’)**, *w;Srp-GAL4,UAS-GFP/UAS-sSpitz^CS^;Crq-GAL4,UAS-GFP/+* **(sSpi^CS^; B, E, G-G’)**, *w;Srp-GAL4,UAS-GFP/+;Crq-GAL4,UAS-GFP/UAS-Spitz^Sec^* **(Spi^sec^; C, F-G’)**, *w;Srp-GAL4,UAS-red stinger/+;Crq-GAL4,UAS-red stinger/+* **(control; H)**, *w;Srp-GAL4,UAS-red stinger/UAS-sSpitz^CS^;Crq-GAL4,UAS-red stinger/+* **(sSpi^CS^; H)** and *w;Srp-GAL4,UAS-red stinger/+;Crq-GAL4,UAS-red stinger/UAS-Spitz^Sec^* **(Spi^sec^; H)**.

**Supplementary Figure 2. Expression of Spitz variants activates ERK signalling in embryonic macrophages *in vivo***

**(A)** Images of single 0.1 μm thick optical sections of GFP-labelled macrophages on the ventral midline at stage 15. Optical sections taken of embryos immunostained for GFP (green in merge) and activated ERK (DpERK; magenta in merge) to show macrophage localisation and as a read-out of EGFR activation, respectively. Top row shows control embryo; middle row shows overexpression of sSpi^CS^; bottom row shows overexpression of Spi^sec^. Scale bars denote 5μm; dotted magenta lines show edge of macrophages in DpERK channels. **(B)** Scattergraph showing quantification of DpERK staining (total intensity per macrophage, per μm^3^, per embryo) in indicated genotypes (n = 3 embryos). Lines and error bars represent mean and standard deviation, respectively. Statistical analysis via one-way ANOVA with a Dunnett’s post-test used to compare control group with Spitz embryos; control vs. sSpi^CS^ *p* = 0.0253, control vs. Spi^sec^ *p* = 0.0016); * and ** represent *p* < 0.05 and *p* < 0.01, respectively**.** Embryo genotypes are as follows: *w;Srp-GAL4,UAS-GFP/+;Crq-GAL4,UAS-GFP/+* **(control)**, *w;Srp-GAL4,UAS-GFP/UAS-sSpitz^CS^;Crq-GAL4,UAS-GFP/+* **(sSpi^CS^)**, *w;Srp-GAL4,UAS-GFP/+;Crq-GAL4,UAS-GFP/UAS-Spitz^Sec^* **(Spi^sec^)**.

## Supplementary Movie Legends

**Supplementary Movie 1. Behaviour of macrophages in control embryos and on macrophage-specific expression of Spitz variants at stage 15 on the ventral midline**

Top row of images corresponds to maximum projections of 1-h long time-lapses of macrophage random migration behaviour at stage 15 on the ventral midline; lower row shows movies with associated tracks. Time-lapses show control embryos (left panel) and those with macrophage specific expression of sSpitz^CS^ (middle panel) or Spitz^sec^ (right panel). Scale bars denote 10 μm. Embryo genotypes are as follows: *w;Srp-GAL4,UAS-GFP/+;Crq-GAL4,UAS-GFP/+* (control, left panel), *w;Srp-GAL4,UAS-GFP/UAS-sSpitz^CS^;Crq-GAL4,UAS-GFP/+* (sSpitz^CS^, middle panel) and *w;Srp-GAL4,UAS-GFP/+;Crq-GAL4,UAS-GFP/UAS-Spitz^Sec^* (Spitz^sec^ , right panel).

**Supplementary Movie 2. Macrophage-specific expression of Spitz variants impairs migration to epithelial wounds at stage 15**

Maximum projections of time-lapse movie of macrophages responding to an epithelial wound introduced via laser ablation at stage 15 on the ventral midline; initial frame shows pre-wound image. Movies show control embryos (left panel) and those with macrophage-specific expression of sSpitz^CS^ (middle panel) or Spitz^sec^ (right panel). Scale bars denote 10 μm. Embryo genotypes are as follows: *w;Srp-GAL4,UAS-GFP/+;Crq-GAL4,UAS-GFP/+* (control, left panel), *w;Srp-GAL4,UAS-GFP/UAS-sSpitz^CS^;Crq-GAL4,UAS-GFP/+* (sSpitz^CS^, middle panel) and *w;Srp-GAL4,UAS-GFP/+;Crq-GAL4,UAS-GFP/UAS-Spitz^Sec^* (Spitz^sec^ , right panel).
